# P37 A rare cause of autoimmune hepatitis secondary to a biological therapy

**DOI:** 10.1093/rap/rkac067.037

**Published:** 2022-09-28

**Authors:** Hashmi Hashim, Afzal Latheef, Rizwan Rajak

**Affiliations:** King's College Hospital NHS Foundation Trust, London, United Kingdom; St George's University Hospitals NHS Foundation Trust, London, United Kingdom; Croydon Health Services NHS Trust, London, United Kingdom

## Abstract

**Introduction/Background:**

Psoriatic arthritis is a heterogenic, immune-mediated musculoskeletal disease that presents with inflammation of joints, enthesis, and axial skeleton, and is associated with cutaneous psoriasis.

Anti-TNF therapy has been known to trigger autoantibody response. Several cases of Autoimmune Hepatitis (AIH) have been reported. Anti-TNFα therapy in AIH is mainly attributed to the disruption of the regulatory role of TNFα signalling on the immune system. Close monitoring is essential to identify any evolving autoimmune events while on TNF antagonists. Treatment requires discontinuing the agent in mild cases and in those with systemic involvement, treatment with steroids and specialist referral is warranted.

**Description/Method:**

This 39-year-old South African origin gentleman was initially referred to the Rheumatology department in 2013 with throbbing pain in his hands and stiffness in heels associated with scalp and nail changes secondary to psoriasis. His past medical history included psoriasis and sports trauma related left anterior cruciate ligament (ACL) tear. He also had a strong family history of psoriasis.

His initial autoimmune results were negative including a negative ANA. The Magnetic Resonance Imaging (MRI) of his right ankle at the time, revealed enthesopathy with surrounding oedema, consistent with psoriatic arthropathy.

The patient was managed initially with methotrexate. Over following months, he experienced significant fatigue and hypersomnia, and stopped the medication. He re-presented one and half years later with a flare on his hands and feet. The clinical examination showed onycholysis, guttate pattern psoriasis and synovitis in his PIPJs. He was started on Leflunomide, and his symptoms improved, however he suffered loose stools. The drug frequency was tapered, but the efficacy was poor leading to recurrent flares.

Subsequently, he was switched to Adalimumab in 2016 and his symptoms improved significantly. However, after a few months his monitoring bloods showed that abnormal Alanine Transaminase (ALT) levels soaring to 263 U/L, with normal albumin, bilirubin and alkaline phosphatase. A repeat autoimmune study showed strongly positive Anti-Nuclear Antibody (ANA) Hep2 cells (1:1280, mixed pattern homogenous and speckled). His Adalimumab was stopped and within 2 months his ANA became negative and ALT normalized. He was then switched to Ustekinumab.

In 2018, he started to notice finger swellings especially on the left hand and ultrasound showed paratenonitis of the left third and fourth proximal interphalangeal joints and synovitis of the left 1st distal interphalangeal joint consistent with active psoriasis. He was then switched to Secukinumab which he has remained on with sustained remission and no adverse effects.

**Discussion/Results:**

We present a rare case of adalimumab induced autoimmune hepatitis in a patient with psoriasis, who developed strongly positive ANA during the course of treatment. Following cessation of adalimumab, his liver profile returned to normal limits and ANA became negative.

Autoimmune hepatitis (AIH) is a chronic inflammation of the liver caused by hepatocyte-specific autoantigens. Not all pathophysiologic mechanisms of AIH are fully understood however, there is emerging evidence that genetic predisposition, molecular mimicry, and an imbalance between effector and regulatory immunity are important factors for disease development and progession. Liver biopsy results for autoimmune hepatitis classically reveal interface hepatitis, focal necrosis, portal inflammation, and plasma cells. Hepatotoxicity is an expected adverse reaction of tumour necrosis factor-α (anti-TNF-α) blocking agents including, infliximab, etanercept and adalimumab. The majority of cases appeared between one month and one year after initiation of the biological agent and complete resolution was observed in nearly 75% of cases after cessation of therapy. Evidence suggests that patients with psoriasis have a higher risk of AIH.

Drug induced AIH and drug induced liver injury (DILI) can be quite challenging to diagnose due to the similar presentations and a recent study also suggest that infliximab was associated with a higher risk of DILI than other agents like adalimumab and etanercept.

Our literature search has shown, that TNF-alpha antagonists, especially adalimumab and infliximab are known to trigger many autoimmune conditions like Psoriasis, Inflammatory Bowel disease (IBD), Systemic Lupus Erythematosus (SLE), and vasculitis, with the majority of cases having good outcomes following cessation of drug. Drug induced demyelinating disease and interstitial lung disease is noted to have poor outcomes. Interestingly, biologic agent induced IBD is more common than AIH.

**Key learning points/Conclusion:**

The number and complexity of drug-induced autoimmune syndromes with biologics has increased in the recent years. Anti-TNF-induced AIH has occurred most commonly with Infliximab and much less commonly with adalimumab or etanercept.

Physicians dealing with patients treated with biological therapies should be aware of the possible development of several autoimmune phenomena, and close follow-up and monitoring is essential.

Before commencing anti-TNF therapy in patients with any autoimmune conditions, it is imperative to have a baseline liver profile and autoimmune profile so as to differentiate between AIH and other causes of liver derangement including genetic predisposition. It is also crucial to rule out other potential causes of hepatitis including alcohol use, viral infection, and hepatotoxic medications.

Clinical response is excellent in almost all cases following discontinuation of anti TNF therapy and in severe cases low or moderate steroid therapy and hepatologist input is needed. Close monitoring and regular follow-up with blood tests would help in minimising adverse drug reactions. Referral to hepatologist is recommended if ALT> 3 times the upper limit of normal or when there is an increase in bilirubin or onset of jaundice.

